# An Epitome of the History of Medicine

**Published:** 1898-04

**Authors:** 


An Epitome of the History of Medicine. By Roswell Park, A. M., M. D., Professor of Surgery in the Medical Department of the University of Buffalo, etc. Illustrated with Portraits and other Engravings. One volume, Royal octavo, pages xiv—348. Extra cloth. Beveled edges. $2.00 net. The F. A. Davis Co., Publishers, 1914 and 1916 Cherry Street, Philadelphia; 117 West Forty-second Street, New York; 9 Lakeside Building, Chicago.

This volume is intended to be a text-book for medical colleges. The author claims that the history of medicine has been too much neglected, and that it ought to be included in the course of study in the colleges. He has therefore prepared this volume, in which the main facts are given with avoidance of too much detail. It therefore may also serve as a book of reference, and even be read by the laity with profit. It is illustrated, also, mainly with copies of old wood-cuts, giving portraits of prominent men and early colleges, and early forms of surgical instruments.

His estimate of Paracelsus is severe, and in the main close to known facts, yet he also says of him: "Few men there are of whom so much good or so much evil has been written as of Paracelsus. Few are there of whom it is to-day so hard to judge, since, if we refer to his contemporaries, they disagree completely concerning him, and if we refer to his own writings we fall into still greater chaos, and have to abandon the attempt. His writings show ideas without connection, observations which contradict each other, and phrases which defy comprehension. At one moment he gives proof of admirable penetration, at the next simply abject nonsense."

This Paracelsus was the man who wrote: "Know, ye doctors, that my hat knows more than you; that my beard is more experienced than your academies. Greeks, Latins, Arabians, French, Italians, Jews, Christians, and Mohammedans, you must follow me; I shall not follow you, for I am your monarch, and sovereignty belongs to me." The full name of the author of these modest pretensions was Aurelius Phillipus Theophrastus Paracelsus Bombastes ab Hohenheim!

The author whose work we are reviewing sums up the value of Paracelsus in these words: "Altho' this man was such a prominent character in his day, his name must be erased from the list of those who have contributed to the world's progress. He was simply a pretended reformer, who counted as nothing the most erudite writings, and who relied solely on his own experiences. He had the most profound self-confidence, and played upon the credulity of his neighbors and victims with the toys which were furnished him by the prevalent cabalistic notions of the day. The school which he would have founded was nothing but a school of ignorance, dissipation, and boasting—a school of medical dishonesty." This account is embellished with a portrait derived from an old engraving of Paracelsus.

On page 225 the author tells the history of the origin of vaccination. According to this statement the principle of "preventive inoculation against small-pox" was known to the ancients, especially the Brahmins. The Chinese, ten centuries before Christ, practiced a modification of the method, using "pledgets of cotton saturated with variolous pus," which were introduced into the nasal cavities of young children. The practice of inoculation was then brought into the west by Lady Mary Wortley Montagu, wife of the English Ambassador to the Porte. The rest of the history is too familiar to need repetition here.

The author notices Homœopathy, and attributes its rise to a reaction against the excessive dosing of the day. He regards it as a rebound to the other extreme of "practical therapeutic nihilism." He says the doctrine of *Similia* was not original with Hahnemann, but had been formulated by Hippocrates, and later by Paracelsus. No further comment on Homœopathy is made, but he notices Isopathy as one of its offshoots, and declares it to be "perhaps the filthiest theory ever invented." He makes a ludicrous error in defining Isopathy as a principle, "according to which like is to be cured by like." Then he notices another "offshoot" of Homœopathy—Mattei's Electrohomœopathy, with its "red," "blue" and "green" electricity, which he rightly denominates "utter idiocy."

A contemptuous notice of Phrenology follows the foregoing, and then he returns to the ordinary developments of the old school.

A high tribute is paid to the genius of Andral, who taught the doctrine of blood dyscrasiæ, and who is memorable for his attempts to disprove the truth of Homœopathy by instituting experiments in the hospitals (under adverse conditions, of course) to show its inefficacy.

Finally, the author notices the University of Pennsylvania at Philadelphia as having the honor of being the first medical school in America. A copy of an old print is inserted, showing the humble beginnings of the University in Fifth Street, this city. We may here digress to speak of the growth of the University of Pennsylvania since its foundation a hundred and twenty years ago. It was removed to Ninth Street, where it was composed of two large buildings—a medical department and an academic department. Later it was removed to West Philadelphia, where it had expanded to four large buildings, valued at half a million dollars. Now, under the wonderful administration of Dr. William Pepper, the Provost, who may well be styled the father of the University, it has expanded to a village of forty-five immense buildings, valued at from ten to fifteen millions of dollars. Lately, Dr. Pepper has resigned the office of Provost in order to devote himself more closely to the medical department, and has named his own successor, Mr. Charles C. Harrison, a well-known business man. In its extraordinary progress, thanks to the rare executive ability of Dr. Pepper, the University is seeking to include in its curriculum every department of human knowledge. We, therefore, confidently expect that it will sooner or later add the teaching of genuine Homœopathy. Efforts in that direction have not been wanting, and they have not been repelled entirely, as would naturally be expected. Hence, we have justification for our expectations in this direction.

